# Impact of Hearing Loss on Memory Loss in Patients With Self-Reported Olfactory Dysfunction

**DOI:** 10.7759/cureus.93357

**Published:** 2025-09-27

**Authors:** Mitali Sakharkar, Asritha Sure, Mingqian Tan, Marianella Paz-Lansberg, Jessica Levi

**Affiliations:** 1 Department of Otolaryngology - Head and Neck Surgery, Boston University School of Medicine, Boston, USA; 2 Department of Otolaryngology and Rhinology, Boston Medical Center, Boston, USA; 3 Department of Otolaryngology and Pediatric Otolaryngology, Boston Medical Center, Boston, USA

**Keywords:** cognitive impairment, dementia, hearing loss, memory loss, olfactory dysfunction

## Abstract

Introduction

The association between hearing loss and dementia, and olfactory dysfunction (OD) and dementia, respectively, is well-elucidated. Several neurodegenerative diseases, including Alzheimer’s disease and Parkinson’s disease, impact the olfactory system early in their progression. Despite these established correlations, the independent effect of hearing loss on memory in patients with self-reported olfactory dysfunction is poorly elucidated, which this study aims to investigate, especially in light of the prevalence of anosmia post-COVID-19.

Methods

A total of 591 records of adult patients with self-reported olfactory dysfunction without a history of traumatic brain injury (TBI) from 2015 to 2020 from a single academic institution were analyzed. Patients were stratified based on demographic, memory loss (including mild cognitive impairment (MCI)), and clinical factors. Univariate and multicollinearity analyses assessed for correlations, with significant covariates utilized in a logistic regression model.

Results

In univariate analyses, hearing impairment (p<0.001), history of stroke (p<0.001), coronary artery disease (CAD) (p<0.001), hypertension (HTN) (p<0.001), and obstructive sleep apnea (OSA) (p=0.020) were significant. Primary language spoken, smoking history, myocardial infarction (MI), and diabetes mellitus (DM) were not significant. In logistic regression, hearing impairment was significantly associated with memory loss (p<0.001). Other significant covariates included stroke (p=0.004), CAD (p=0.022), and HTN (p<0.001).

Conclusions

Our results show that hearing impairment is significantly associated with memory loss, specifically in patients with self-reported olfactory dysfunction. This highlights the increased risk of memory loss in this population, underscoring the need for further research into screening and pathophysiology of memory loss in hard-of-hearing patients with olfactory dysfunction post-COVID-19.

## Introduction

Hearing loss, anosmia, and dementia are increasingly prevalent conditions in the USA with interconnected pathophysiologies. Approximately 15% of American adults experience some degree of hearing loss, with the prevalence rising significantly among individuals aged 65 and older. Etiologies of hearing loss are multifactorial, but predominantly sensorineural, with risk factors including aging, noise exposure, and chronic medical conditions, including diabetes and cardiovascular disease [1]. Pre-COVID-19, partial and complete anosmia were estimated to affect 19.1% of the population [2]. In light of the COVID-19 pandemic, anosmia, or the loss of the sense of smell, has become more prominent, with more than 50% of COVID-19+ individuals experiencing ongoing olfactory dysfunction (OD) months after acute recovery [3]. Approximately 60% of COVID-19+ individuals have experienced partial or complete OD, and persistent anosmia rates following upper respiratory infections (URIs) are estimated to be 10% in the USA [2,4]. Lastly, memory loss, from mild cognitive impairment (MCI) to neurodegenerative dementias, impacts an estimated 20%-30% of individuals aged 65 and older in the USA and is projected to increase as the global population ages [5].

Hearing loss has been identified as a significant modifiable risk factor for dementia and has been linked with increased cognitive load. The additional mental resources needed to process auditory cues in individuals with hearing loss typically come at the expense of bandwidth for non-auditory cognitive functions [1,6]. Additionally, given that hearing loss can lead to social isolation due to the inability or difficulty comprehending verbal communication, this can exacerbate the underlying risk of dementia psychosocially by reducing opportunities for cognitive stimulation [7,8]. Furthermore, Erkkinen et al. demonstrated neuroanatomical differences in individuals with hearing impairment at baseline, including reduced brain volume in auditory and cognitive regions secondary to neural pruning mechanisms [9,10]. However, despite these theories, the quantitative relationship between the degree of memory loss and neurodegeneration due to hearing impairment is still not fully elucidated.

Sensory dysfunctions have been noted to have impacts on neural well-being [11]. In particular, OD has been recognized as a robust early marker of neurodegenerative diseases, such as Alzheimer’s disease and Parkinson’s disease [12]. Among individuals with normal cognitive function at baseline, any OD is linked to an increased risk of developing dementia [13-15]. In addition, OD can strongly predict the risk of conversion from mild cognitive impairment to dementia, with an estimated risk increase exceeding 80% between cohorts with increasing subjective degrees of OD [16,17]. Anosmia, a more severe form of OD, is associated with a higher risk of developing dementia compared to milder conditions, especially among men and individuals aged 78 and younger [13]. Although it remains unclear whether anosmia is a causative factor or an early symptom of neurodegeneration, many proposed explanations of this pathology highlight increased underlying risks even in individuals below the subclinical threshold [13,18,19]. This underscores the need for research into the shared pathophysiology and management strategies for dual olfactory and cognitive impairment.

The independent relationships between hearing loss and dementia, as well as anosmia and dementia, are well-established. However, the interaction between hearing loss and anosmia in the context of cognitive decline has not yet been thoroughly investigated. Although it is well-known that sensory losses increase the risk of neurodegeneration, the interaction between hearing loss and anosmia in the context of cognitive decline has not been thoroughly investigated. Especially in light of the increased prevalence of post-COVID-19-related anosmia, it is increasingly pertinent to explore the risk factors that may elevate the likelihood of developing dementia in patients with anosmia. As hearing loss is an established, modifiable risk factor for dementia, its potential role as an additional disability in this context deserves further exploration. By elucidating the shared pathophysiology and possible superimposed risk for cognitive impairment, screening interventions targeting at-risk populations can be conducted. In this study, we will explore hearing loss as a potential risk factor for the increased risk of developing dementia in patients with established anosmia.

## Materials and methods

Data source and participants

This study was deemed exempt by our local institutional review board, and a waiver of informed consent was granted.

A retrospective study was conducted at our single academic research hospital. A total of 1,369 adult patients who presented to their primary care providers or ENT clinic from 2015 to 2023 for evaluation for self-reported OD were de-identified, and their charts were reviewed. Inclusion criteria included patients presenting with clinical concern for OD (including anosmia or hyposmia). Exclusion criteria included patients with a history of traumatic brain injury (TBI). For patients with multiple evaluations for self-reported OD, only the first visit was included. All participant information was de-identified for analysis.

Primary exposure and primary outcome variables

The primary exposure was a history of hearing impairment. The primary outcome was memory loss, defined by extracting relevant Current Procedural Terminology (CPT) codes encompassing memory loss, including the spectrum from mild cognitive impairment to dementia. All CPT codes were manually re-chart reviewed as well to ensure that memory loss was appropriately coded and evaluated by two authors (AS and MT). Any discrepancies were resolved by author MS.

Covariates

Additional demographic information included age, sex, body mass index (BMI), race/ethnicity, preferred spoken language, current marital status, and insurance status. Medical comorbidities included were hearing impairment (defined by noted need for hearing aids or inclusion on problem list), diabetes, hypertension (HTN), chronic obstructive pulmonary disease (COPD), cerebrovascular accident (CVA), myocardial infarction (MI), stroke, congestive heart failure (CHF), coronary artery disease (CAD), gastroesophageal reflux disease (GERD), rhinitis, dysphagia, hearing loss, obstructive sleep apnea (OSA), head and neck cancer, memory loss, traumatic brain injury (TBI), upper respiratory infection (URI), and chronic kidney disease (CKD). All medical comorbidities were represented as binary variables, with either the presence or absence of any of the medical comorbidities listed above, as determined by the problem list and/or history and physical documentation at the time of the last visit for OD.

Statistical analysis

Subjects with missing data for the exposure and outcome of interest were excluded. Other variables with missing data containing those variables were excluded from analyses. Data were described using counts and percentages for categorical variables, and medians and interquartile range (IQR) for continuous variables. Non-parametric tests were utilized to account for the lack of normal distribution of exposure and outcome variables. Comparisons between groups were performed using the Mann-Whitney U test for continuous independent variables, the Chi-squared test for categorical independent variables, and Fisher’s exact test for binary independent variables [5,16]. Test statistics are reported for the Mann-Whitney U test (U) and Chi-squared test (χ²) [4,16,20]. Fisher’s exact test does not include a test statistic. Statistical significance was defined as a p-value of less than 0.05. Statistical analysis was completed with SAS version 9.4 (SAS Institute, Inc., Cary, NC).

Unadjusted analysis

First, covariates were analyzed for their associations with OD. Utilizing memory loss as a binary variable, covariates were analyzed using the Mann-Whitney U test and Fisher’s t-tests for continuous and binary covariates, respectively. To evaluate for potential modifiers or confounders, an additional unadjusted analysis was conducted to evaluate the relationship of our covariates. This analysis was completed with a multicollinearity analysis using the variance inflation factor, as appropriate. Variables with a p-value of <0.05 on any of the unadjusted analyses were included in the final model.

Adjusted analysis

A multivariable logistic regression model was applied to determine the association between hearing impairment and memory loss, controlling for all relevant covariates identified on unadjusted analysis. Relevant covariates identified on unadjusted analysis were set by a cut-off of p<0.05.

## Results

Sample selection

A total of 591 unique patient records were returned by our original inclusion and exclusion criteria.

Records with their first clinic visit date for evaluation for OD were reviewed. Missing data included primary language spoken (n=4, 0.29%), race (n=219, 15.9%), and marital status (n=40, 2.9%) (Table 1).

**Table 1:** Demographics of Patients With and Without Memory Loss *p<0.05 **p<0.01 ***p<0.001 This table presents demographic characteristics and the results of univariate analyses stratified by patients with and without memory loss. Categorical variables were compared using Fisher’s exact test, which does not produce a test statistic. Continuous variables were compared using the Mann-Whitney U test, with a reported test statistic. SD: standard deviation, MI: myocardial infarction, HTN: hypertension, CHF: congestive heart failure, DM: diabetes mellitus, CAD: coronary artery disease, OSA: obstructive sleep apnea, CKD: chronic kidney disease

Variable	Memory loss (N=52)	No memory loss (N=539)	p-values (test statistic)
Age (years)			<0.001*** (U=1,872,783)
Number	52	539	
Mean (SD)	67.8 (12.30)	46.6 (15.02)	
Median	69	47	
Q1-Q3	58.5-76.5	36-57	
Min-max	30-93	18-86	
Sex			1
Male	21 (40.4%)	218 (40.4%)	
Female	31 (59.6%)	321 (59.6%)	
Race			0.65
White	15 (34.9%)	119 (30.4%)	
Black or African American	19 (44.2%)	193 (49.2%)	
Asian	1 (2.3%)	24 (6.1%)	
Other	8 (18.6%)	56 (14.3%)	
Ethnicity			0.51
Hispanic or Latino	12 (24.0%)	152 (29.1%)	
Not Hispanic or Latino	38 (76.0%)	370 (70.9%)	
Primary language			1
Non-English	20 ( 38.5%)	204 (38.0%)	
English	32 (61.5%)	333 (62.0%)	
Hearing impairment			<0.001***
Yes	22 (42.3%)	60 (11.1%)	
No	30 (57.7%)	479 (88.9%)	
Smoking history			0.55
Yes	46 (88.5%)	453 (84.0%)	
No	6 (11.5%)	88 (16.0%)	
MI			0.15
Yes	50 (96.2%)	481 (89.2%)	
No	2 (3.8%)	58 (10.8%)	
HTN			<0.001***
Yes	44 (84.6%)	202 (37.5%)	
No	8 (15.4%)	337 (62.5%)	
CHF			<0.001***
Yes	8 (15.4%)	15 (2.8%)	
No	44 (84.6%)	524 (97.2%)	
DM			<0.001***
Yes	25 (48.1%)	131 (24.3%)	
No	27 (51.9%)	408 (75.7%)	
CAD			<0.001***
Yes	13 (25.0%)	23 (4.3%)	
No	39 (75.0%)	516 (95.7%)	
OSA			0.020*
Yes	10 (19.2%)	45 (8.3%)	
No	42 (80.8%)	494 (91.7%)	
Stroke			<0.001***
Yes	7 (13.5%)	7 (1.3%)	
No	45 (86.5%)	532 (98.7%)	
CKD			0.006**
Yes	13 (25%)	58 (10.8%)	
No	39 (75.0%)	481 (89.2%)	
Marital status			0.54
Yes	33 (63.5%)	362 (67.5%)	
No	19 (36.5%)	174 (32.5%)	
Private insurance			<0.001***
Yes	158 (40.5%)	232 (59.5%)	
No	3 (6.8%)	41 (93.2%)	

Sample characteristics

Overall, the cohort was largely women (n=797, 58.2%) and non-white (n=726, 68.2%). Of the cohort, 622 (45.4%) patients were diagnosed with olfactory dysfunction, 112 (8.2%) had memory loss, and 155 (11.3%) had a diagnosed hearing impairment.

In patients with OD and without a history of TBI, the cohort was largely women (n=352, 59.6%) and non-white (n=300, 69.1%). Of the cohort, 52 (8.8%) had memory loss, and 82 (13.9%) had a diagnosed hearing impairment.

Unadjusted analysis

Hearing impairment (p<0.001), a history of stroke (p<0.001), coronary artery disease (CAD) (p<0.001), hypertension (HTN) (p<0.001), and obstructive sleep apnea (OSA) (p=0.02) were significantly associated with memory loss (Table 1). Sex, race, ethnicity, primary language, smoking history, history of myocardial infarction (MI), and marital status were not associated with memory loss (Table 1).

Adjusted analysis

On adjusted analysis, a logistic regression analysis was performed with all covariates with a p<0.05. A multicollinearity test was run using the variance inflation factor to test for any interaction between covariates. No significant interaction was found among covariates.

**Figure 1:**
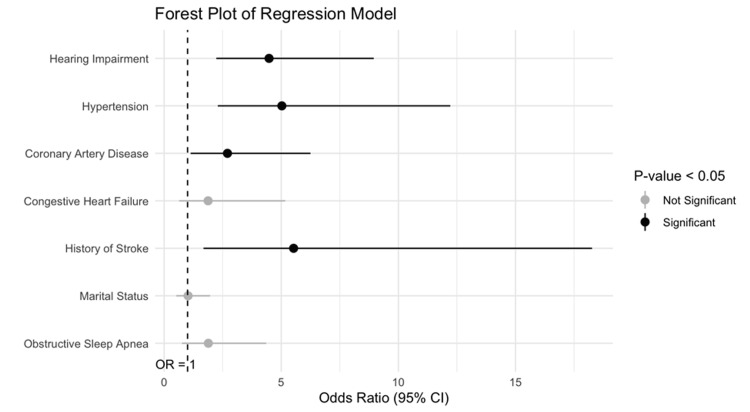
Forest Plot of Regression Model Figure 1 displays a forest of OR of the final regression model of hearing impairment on memory loss. The dashed line represents an OR of 1.0. Grey lines represent non-significant covariates, and black lines represent significant covariates. OR: odds ratio, CI: confidence interval

On multivariate logistic regression, in patients with OD, hearing impairment was significantly associated with memory loss (p<0.001). Other significant covariates included stroke (p=0.004), CAD (p=0.022), and HTN (p<0.001) (Figure 1). To ensure the model was not overfit, a 10-fold internal validation bootstrap analysis was performed, with an average area under the curve (AUC) of 0.71. Odds ratios and confidence intervals for the final model are provided in Table 2.

**Table 2:** Odds Ratios and Confidence Intervals for Final Regression Model This table displays the final regression model’s odds ratios and confidence intervals on memory loss.

Covariate	Odds ratio	Confidence interval
Hearing impairment	4.48	2.25-8.93
Hypertension	5.02	2.20-11.4
Coronary artery disease	2.70	1.16-6.29
Congestive heart failure	1.88	0.67-5.26
History of stroke	5.53	1.71-17.9
Marital status	1.02	0.52-2.00
Obstructive sleep apnea	1.89	0.80-4.47

## Discussion

**Figure 2:**
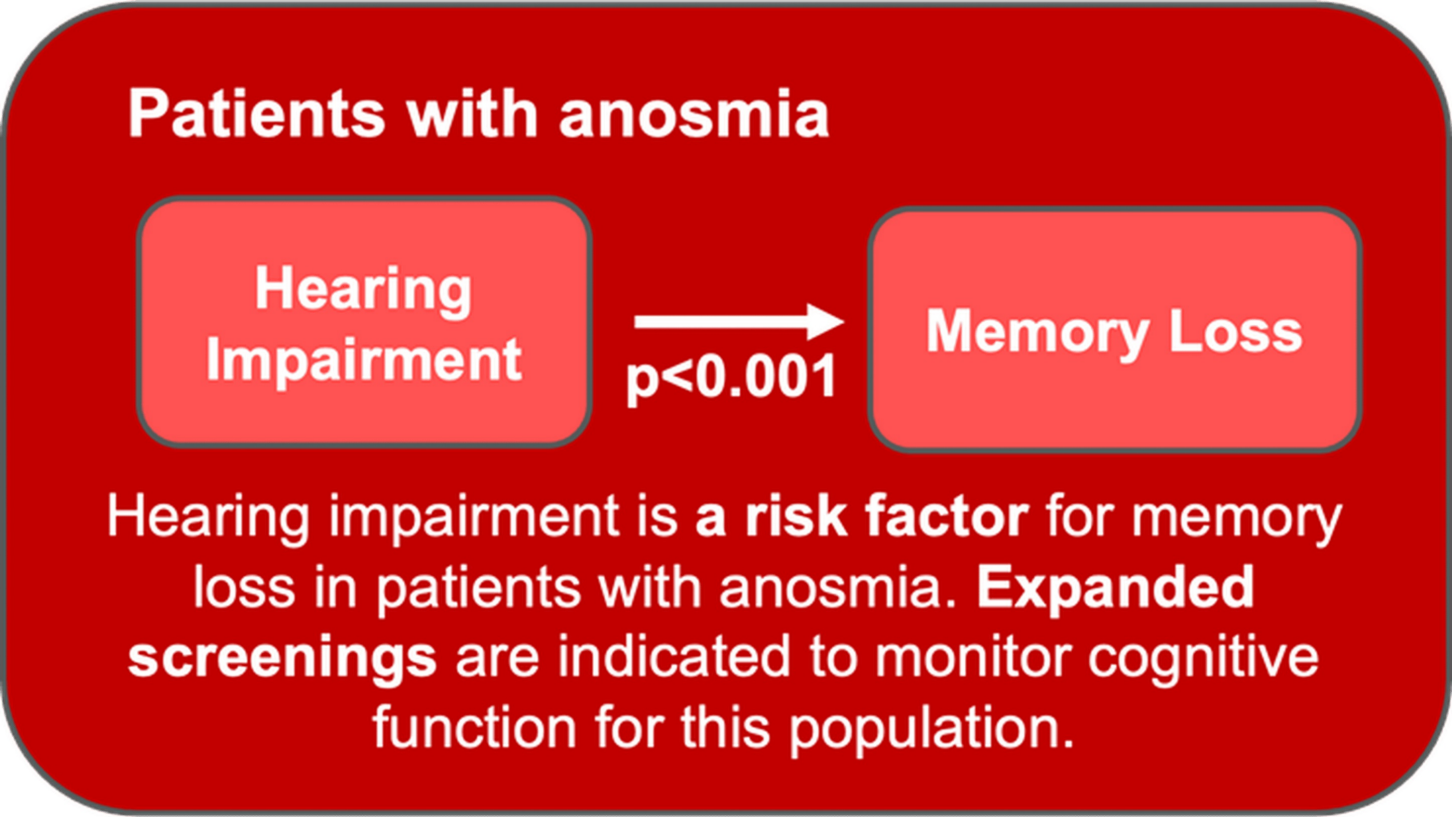
Schematic of Hearing Impairment, Memory Loss, and Anosmia Figure 2 displays a pictorial schematic of the interplay of anosmia, hearing loss, and memory loss as a synopsis statement.

To the best of our knowledge, this study is the first to investigate the association between hearing loss and memory loss in patients with OD. In line with our hypothesis, we found that a history of hearing impairment was associated with greater odds of memory loss in patients with OD (Figure 2).

Our data provides important context to prior literature describing the unique risk of hearing loss in patients with impaired smell and memory. This study demonstrated that dual sensory deficits may have a greater impact on morbidity than previously acknowledged. Our results demonstrate that hearing loss in patients with anosmia increases the risk of developing memory loss-related conditions, such as cognitive impairment and dementia. Literature explanations of prior proposed mechanistic relationships between hearing loss and increased risk of dementia may be explained by three factors: decreased hippocampal and entorhinal cortex volume, reduced socialization, and increased cognitive load associated with living with hearing loss [1,7,8]. Individuals with pre-existing OD are already at an elevated risk of developing dementia, likely driven by neurodegenerative pathological processes, such as protein buildup in the olfactory bulbs and tracts [21]. Thus, as supported by our findings, our addition of hearing loss may compound this risk by further increasing cognitive load, intensifying social isolation, and exacerbating functional limitations that reduce physical activity. Together, these factors may create a cumulative effect, significantly raising the likelihood of cognitive decline and dementia [22].

Furthermore, sensory deficits are likely to be significantly underreported. Watson et al. demonstrated that even COVID-19+ individuals with anosmia are often unaware of their sensory deficits, leading to massive underreporting [23]. Studies indicate that less than one-third of those with OD recognize their impairment, while nearly one-third of individuals with hearing loss are unaware of their condition [24,25]. Even when raised to the threshold of clinical attention, self-reports of these conditions consistently underestimate the extent of the impairment when compared to objective measures, such as audiology and olfactory tests [15,24,26]. This especially holds true among older adults, potentially due to individuals adapting to their hearing loss over time or considering it an inevitable aspect of aging [24]. This low sensitivity of self-reports for OD is due to a general lack of awareness, even in well-educated and health-conscious populations [25]. Given the findings of this study, it is critical that the underlying hearing loss is mitigated with appropriate interventions, especially in populations with numerous sensory deficits.

As demonstrated previously, hearing loss and anosmia have both been independently associated with an increased risk of developing dementia. Our study has demonstrated that hearing loss in patients with anosmia is linked to a greater association with memory loss-related conditions. Therefore, this paper adds to the literature on the impact of dual sensory deficits, which may be crucial for informing early interventions and tailored management strategies. Examples of early interventions include hearing aids and olfactory training, which have been shown to improve olfactory function and potentially mitigate cognitive decline by enhancing neural plasticity [27,28]. Furthermore, early screening interventions for sensory losses have been shown to successfully identify subclinical hearing loss/olfactory deficits and prevent delays in diagnosis and an increase in morbidity that would otherwise result from the time for deficits to rise to clinical and the patient’s attention (Figure 2) [29]. It is critical to implement screenings for individuals using validated objective tests. Examples of such include the HearCheck Screener and the Pennsylvania Smell Identification Test [30].

However, the study also has multiple limitations. This study has a relatively small sample size and was conducted at a single institution. Hearing and olfactory tests were not directly administered; instead, we relied on retrospective chart review to inform the presence of sensory deficits. Additionally, the study used a CPT code to identify memory loss-related conditions, which range from mild cognitive impairment to dementia, instead of using a standardized cognitive assessment tool such as the Montreal Cognitive Assessment (MoCA). All CPT-extracted data variables were cross-verified by manually parsing through patient problem lists and noted diagnoses to verify the accuracy of the diagnosis. Furthermore, no objective measure of olfactory dysfunction was utilized; as such, this study is limited by self-reported concerns of OD. Despite manual review of all CPT codes, the risk of misclassification cannot be ruled out, especially given the self-reported nature of data variables. However, prior literature evidence demonstrated that self-reported OD was often underreported in comparison to objective OD. Additionally, since our patient sample was limited to a single institution, expanding inclusion criteria to multiple institutions and geographic locations could increase the generalizability of these results. Lastly, this study is limited by the retrospective nature of its design. As a result, the relationship between the variables time and courses may not be well-elucidated, and causal inference is limited.

Future research should aim to validate these findings in a prospective design with questionnaires to standardized hearing loss, anosmia, and cognitive impairment screenings, and determine mechanisms that mitigate this relationship. Interventions that could help in mitigating risks include increased screenings for patients who present for COVID-19 or present with a decreased sense of smell to undergo audiology at that appointment as well.

## Conclusions

Overall, this study demonstrates an association between the presence of hearing impairment and memory loss in patients with self-reported olfactory dysfunction. Our results show that hearing impairment may be significantly associated with memory loss on retrospective analysis, specifically in patients with self-reported olfactory dysfunction. This may highlight the increased risk of memory loss in this population and suggest the need for further research into screening and pathophysiology of memory loss in hard-of-hearing patients with olfactory dysfunction, especially post-COVID-19, as well as prospective studies with objective questionnaires to explore the potential of causal relationships further.
